# Current psychopathology models emphasize very early intersubjectivity-based interventions in children to prevent later mental disorders

**DOI:** 10.3389/fpsyg.2023.1225108

**Published:** 2024-01-24

**Authors:** Lisa Ouss

**Affiliations:** Child and Adolescent Psychiatry Department, Necker-Enfants Malades Hospital, APHP, Paris, France

**Keywords:** psychopathology, development, interventions, classifications, P factor, phenomenology, psychodynamic, intersubjectivity

## Abstract

Current psychopathology models have evolved toward dimensional models, in which symptoms and diseases are at the extremes of dimensions. Despite these new dimensional proposals, classifications and third-person approach have shown limitations. Their extraordinary evolution nevertheless underlines the contributions of developmental and psychodynamic frameworks. Developmental contributions have made it possible to evolve from disorders centered on a first-person perspective. Complementarily to the first-person/third-person perspectives, we advocate a second-person perspective, based on intersubjectivity. This perspective reverses the intuitive trend to focus our interventions on the most specific symptoms and syndromes, and advocates instead interventions on a “p” general factor that are both generalized and highly targeted. The implications are (1) to intervene as early as possible, (2) to base the definition of our therapeutic targets on an intersubjective perspective, (3) to identify and enhance children’s and parents’ strengths. These empirically informed directions are not in the current mainstream of psychopathology frameworks, and need to be developed.

## Psychopathology models have evolved toward dimensional models, in which symptoms and diseases are at the extremes of dimensions

Developmental disorders force us to revise our models of psychopathology, which have been essentially built on those of adult psychiatry. The etymological conception of psychopathology, which refers to “psyche/pathos/logy,” aims at the study of pathological manifestations of the human psyche. Psychopathology remains first and foremost the study of pathological human behavior. Its object is the human psyche, i.e., a complex epistemic object whose ontological referent belongs to several fields. We have to combine two approaches: the “nomothetic” one, which aims to determine universal laws, and the “idiographic” one, which grasps the particular (Lyon et al., 2017). Different functions have been proposed for psychopathology: the descriptive function deals with the phenomenological experience of psychopathological suffering, the clinical one organizes the classification of this experience, while the theoretical one explores the etiology of this experience. In the APA dictionary of psychology,^1^ however, the term “Psychopathology” is defined more narrowly as (1) the scientific study of mental disorders (2) the behavioral or cognitive manifestations of such disorders. The term in this sense is sometimes considered synonymous with mental disorder itself. Yet, there are three ways to assess mental illness (Fuchs, 2010): the positivistic, or 3rd-person approach, using classifications; the phenomenological, subject-oriented or 1st-person approach; and the hermeneutic, intersubjective or 2nd-person approach. We will first develop the interest and limits of the first approach. Although alternatives to categorical diagnoses have been proposed, they do not provide access to a person’s experience. We will see that the question of development has paved the way for the second approach. We will finally show how, complementarily, the first two approaches support the absolute necessity of the third one. The implications for early intersubjective interventions will be developed.

## Despite new dimensional proposals, classifications, and third-person approaches have limitations

ICD and DSM classifications are unavoidable tools for scientific communication, but they have limitations. They do not reflect the continuous process of psychopathology, but rather its final categorical manifestation (Markon and Krueger, 2005). The diagnostic categories have sometimes been built top-down, and have changed according to theoretical *a priori*, or to the influence of lobbies in order to allow insurance companies to refund the cost of treatments. They do not take into account the developmental continuity between disorders nor the frequent comorbidities (Caspi et al., 2020). Subthreshold symptoms in individuals with other disorders are not taken into account. Lastly, clinicians are encouraged to diagnose several comorbidities, using the “not otherwise specified” label, in order to facilitate access to treatment (Pincus et al., 2004).

New proposals have revisited the classification into discrete disorders. The first dimensional categorization was proposed in children by Achenbach (1966) and Achenbach and Edelbrock (1978), focusing on patterns of co-occurrence among common psychopathological syndromes in childhood, distinguishing internalizing and externalizing dimensions.

The Research Domain Criteria (RDoC) framework (Insel et al., 2010) was created as an alternative to diagnosis, and is devoted to informing future classifications, and to understanding the transdiagnostic biobehavioral systems underlying psychopathology. The RDoC framework is operationalized in the RDoC matrix, built with eight columns representing units of analysis (genes, molecules, cells, circuits, physiology, behavior, paradigms, and self-reports), and six rows representing functional higher-level domains: Negative Valence Systems, Positive Valence Systems, Cognitive Systems, Systems for Social Processes, Arousal/Regulatory Systems, and Sensorimotor Systems, each domain containing 3 to 6 constructs. All these dimensions were defined “top-down,” based on expert consensus from relevant fields.

The Hierarchical Taxonomy of Psychopathology (HiTOP) alternative (Kotov et al., 2017) is a dimensional classification system, based on observational clinical assessments. It offers a hierarchical organization, from general functioning, to spectra, subfactors and symptoms, which distinguishes traits and syndromes. A general factor is at the top of the system, then increasingly detailed dimensions (internalized, externalized…), conceptualized as extremes of normal psychological functions rather than as categories, represent a continuous spectrum of risk and severity. Caspi and colleagues (Caspi et al., 2014) proposed that common categorical diagnoses of adult psychopathology could be best explained and structured by a general psychopathology latent factor alongside unique internalizing and externalizing latent factors. Covariation among symptoms of psychopathology and maladaptive traits define clusters. The treatment decision-making of clinicians is more aligned with the HiTOP description than with traditional diagnoses (Hopwood et al., 2020). In view of the respective limitations of these two models, it has been proposed that RDoC and HiTOP should be complementary models: RDoC may help elucidate the underpinnings of the clinical dimensions included in HiTOP, while HiTOP may provide psychometrically robust clinical targets for RDoC-informed research (Michelini et al., 2021).

Another alternative, bottom-up built, considers that psychiatric disorders can best be viewed as sets of symptoms that are connected through a system of causal relation. “Symptoms of psychopathology are causally connected through myriads of biological, psychological and societal mechanisms [...] The network theory holds that this is a general feature of mental disorders, which can therefore be understood as alternative stable states of strongly connected symptom networks” (Borsboom, 2017). This new alternative (Borsboom and Cramer, 2013) has led to a new way of conceiving of psychopathology and of answering the question: “What kind of things are psychiatric disorders?” (Kendler et al., 2011). These models do not consider that kinds exist whether or not we recognize them; they “are defined not in terms of essences but in terms of complex, mutually reinforcing networks of causal mechanisms” (Kendler et al., 2011). These Network-based methods are becoming increasingly widespread (Barabási et al., 2011). In the end, however, while we need models to describe the biobehavioral systems underpinning psychiatric disorders, none can avoid the question of the descriptive theoretical context of the basic symptom, which is theoretically and conceptually influenced.

## The evolution of adult models of psychopathology thanks to developmental contributions

Most of the models of psychopathology described above make little reference to development and developmental trajectories. Yet, the main preoccupation of clinicians is to understand when (and why) a psychopathological trajectory begins, and how we can prevent it. Development is a trajectory, and atypical development is a trajectory that results from atypical constraints over time which may have cascading effects on how other skills are acquired (Thomas and Baughman, 2014). To capture atypical profiles, we need developmental and dynamic assessments that draw “trajectories.” Yet, the above-mentioned models seldom refer to time-scales. Measuring trajectories requires the repetition of assessment points in each individual, which is time-consuming and costly. By contrast, methods such as cross-sectional studies that study the differences by comparing assessments at certain points in development detect large-scale associations but do not inform on the idiographic dimension of individual trajectories.

This question concerns not only the appearance of disorders but also the organization of personality traits. Are they precursors of disorders, and if so, how does the interaction between them evolve during development? An interesting review (Durbin and Hicks, 2014) questions the theoretical background of relationships between traits or personality, and disorders. “Trait-disorder associations are dynamic in that their mechanisms differ across persons depending upon their developmental contexts, and within person, based on the idiographic histories of their traits and experience with disorder” (Durbin and Hicks, 2014). Periods of developmental tasks or transitions, such as the transition from adolescence to young adulthood, are especially at risk for these changes. These questions thus require a developmental framework such as that proposed by developmental psychopathology, which assumes that deviations from normal development are likely to signal psychopathological conditions (Cicchetti, 1993). These deviations in trajectories are particularly relevant as first manifestations of a disorder, and as targets for interventions.

## Developmental considerations make it possible to evolve from a disorder-centered to a first-person perspective

How do we determine the aim of our therapeutic interventions? If we follow our two assumptions, that models are now more dimensional, and that they are developmentally informed, we need to determine the following: what is the focus of our interventions? Does the intervention concern a disorder? Or a trait, before the appearance of a disorder? An individual suffering? How can the limit between normal and pathological functioning be identified if all the dimensions assessed are continuous? A disorder arises when it causes the subject problems. Sometimes, however, the subject does not complain, or is not able to complain, for instance due to the person’s developmental stage, as in infants.

All the psychopathological models we have presented are disorder-centered, ranging from normality to pathology. None of them is person-centered, even if network models make it possible to describe individual clusters. None of them refer to phenomenology, only at best to the subject’s inner experience. The terms of phenomenology and experience tend to surreptitiously disappear from the field of psychopathology. The interest in phenomenology, which has progressively lost ground, was recently underlined in psychotherapy practice (Stanghellini and Cravaro, 2014). Our aim is not to develop the concept of phenomenology, but to see how developmental and diachronic frameworks have re-actualized this first-person approach.

De Ajuriaguerra (1989), introducing the developmental approach, argued that the constraint/freedom relationship changes diachronically from birth to adulthood and synchronically during the developmental stages. The constraint/freedom dualism means that the biological equipment constrains the function, but that the functioning of the function, which broadens the degree of freedom, depends more on the way the subject self-organizes the functioning of his/her biological skills. A different way to consider psychopathology would be to take into account both functions (determined by genetic, neurophysiological, and cognitive equipment, occurring in a particular environment at a particular moment), and the functioning of these functions (determined by psychological dimensions, in a social and cultural environment). This “complementarist” approach (Devereux, 1972) would make it possible to avoid opposing the various interpretations readings and interventions. While the HiTOP and RDoC models are interesting in that they distinguish these different levels, neither of them takes into account the way the subject copes with a dysfunctional function. The subject determines how he/she makes the function operate, while the clinician’s role is to determine to what extent and how the function is impaired, but also how the dysfunctioning impairs, or not, the interactive and inner world of the subject. We refer to Ey’s “organodynamism” (Ey, 2006), which “substitutes for monism or dualism the idea of a living dialectic between the vital infrastructure and the psychic superstructure of the person.” It dialectically combines the negativity of the psychopathological process (the pathogenic organic action) with the positivity of the symptoms (the psychic reaction to this action). Therefore, the psychopathological process can hinder being-in-the-world by affecting the synchronic field (the lived experience), as well as the diachronic field of the person (by affecting the progressive integration of these experiences). Mental illness could thus be conceptualized as the knitting together of a vulnerable self, even due to genetical or developmental reasons, and the way a person tries to cope with. Narratization, rather than semi-structured interviews, allows patients to communicate and explain their own experiences, in their own terms, in the context of their personal world and history, and to try to make sense of them, through the method of ‘phenomenological dissection’ (Stanghellini and Cravaro, 2014). This involves a shift away from disease-and-variable-oriented strategies, toward person oriented research and treatment strategies (Bergman and Magnusson, 1997). The person-centered perspective, along with network models, emphasizes the role of multifinality (a given factor may result in a variety of outcomes) and equifinality (there are many pathways toward one specific outcome) (Cicchetti and Rogosch, 1996). The psychodynamically based classification PDM (Lingiardi and McWilliams, 2017) aims to bridge the gap between the need for experimental and methodological validity, and clinical complexity. It attempts to “characterize an individual’s full range of functioning − the depth as well as the surface of emotional, cognitive and social patterns” (Lingiardi and McWilliams, 2017). Nevertheless, this manual, despite its assessment of mental functions in the M axis, remains a categorical classification.

## Towards a second person perspective in interventions, implying targeting a non-specific p factor

The extraordinary shift from a categorial conception of psychopathology toward dimensional and developmental ones completely transforms our field. We propose a hypothesis: the second-person perspective, established through the interaction between a clinician and the patient, offers the most comprehensive understanding of psychopathological processes, the consequent effects of the primary dysfunction, and how patients cope with it. Primarily, this perspective facilitates the development of targeted interventions. But these three perspectives are complementary: the second-person perspective is rooted in the first-person perspective (requiring access to the patient’s subjective experience) and should be interconnected with the third-person perspective (to better delineate the patient’s symptoms and the specific psychiatric treatments needed). The framework of psychopathology should be not monadic unity, but rather dyadic unity: child–parent, child-therapist, parent-therapist, patient-therapist interaction, environment-patient interaction.

But we must go further. We have to reappraise psychotherapy as a highly and precisely targeted intersubjective action. There is a discrepancy between the quantity of knowledge we possess about psychiatric disorders, and the interventions that are effective. A task force (Wampold and Imel, 2015) concluded that “adapting psychological treatment (or responsiveness) to transdiagnostic client characteristics contributes to successful outcomes at least as much as, and probably more than, adapting treatment to the client’s diagnosis.” What works are “transtheoretical common factors” of psychotherapy, that should reduce specific symptoms through their impact on the general factor of psychopathology, and not theoretical ones (Norcross and Wampold, 2018). This conclusion could seem disappointing, but is in fact very important. A very interesting proposal was made by Forbes and colleagues (Forbes et al., 2019). “If all forms of common psychopathology are connected with a general underlying factor that can be observed from the very earliest years of development, then understanding the psychological nature of that general factor […] may provide new directions in contemplating how to reduce levels of the general factor and subsequently prevent a wide range of mental disorders from emerging later in development.” In infants and children, specific psychopathological symptoms are less common: infants often express undifferentiated behaviors not yet organized as patterns. Over developmental time, attractors represent recurrent patterns that have stabilized and are increasingly predictable (Granic and Hollenstein, 2003). We have to intervene before these patterns are installed, and thus target non-specific behaviors. Intervening at the very early roots of developmental tasks in a dimensional perspective, and focusing on this general factor could prevent many later pathological traits and specific symptoms in children and adolescents. Forbes et al. (2019) go further and propose “[using] early intervention for general psychopathology as a foundational scaffold on which to introduce gradually more focused interventions later in development.” This view breaks the dichotomy between prevention, which usually focuses on reducing the first signs or stages of psychopathology, and interventions, which target psychopathological patterns or symptoms. In an attempt to reach this early and non-specific p factor, Fonagy and Allison (2014) proposed that the “p factor may be a proxy for impairments in epistemic trust,” that is, trust in the authenticity and personal relevance of interpersonally transmitted knowledge. For Fonagy and Campbell (2017), psychopathology might be characterized by a temporary or permanent disruption of epistemic trust and the social learning process that it enables. This may have major consequences for future interventions: the p factor could be a “reachability” factor of the patient, and the interventions should be interpersonal or intersubjective ones. The core principles of intersubjectivity, emphasizing the importance of understanding subjective experiences and interactions, are not limited by age. Thus, intersubjective-based interventions can be applicable across various age groups, including youths and adults.

## When to intervene? Critical periods support early interventions

When to intervene? The early beginning of trajectories implies targeting our interventions as early as possible to prevent later psychopathology. The role of the clinician could be to identify targets and intervene at different levels simultaneously: at the higher level of general functioning, targeting the roots of multiple dysfunctions, but also at lower levels, aiming to reach a specific symptom or behavior, with a specialized intervention. These two types of intervention become complementary rather than competing.

But when does the “early” time for intervention begin? While there is a legitimate focus on determining the emerging signs of developmental disorders as soon as possible, how can a disorder be determined before the scheduled appearance of the function that is assumed to be impaired? Is it possible to determine an optimal moment to undertake interventions? The notion of sensitive/critical periods gives some insights into the early processes that underline pattern construction.

A critical period (CP) refers to a finite period in which experience provides information that is essential for normal development and alters performance permanently (Knudsen, 2004). A sensitive period refers to a period in which the effect of experience on the brain is particularly strong during a limited period in development. If experiences essential to cortical specialization do not occur during the CP, the functioning of the cortical areas allocated to the particular skill will be altered, without residual plasticity; if they do not occur during the sensitive periods, it may be difficult to redirect development along a typical trajectory; plasticity exists but to a limited degree. These two concepts refer to two mechanisms. Experience-expectant mechanisms (Greenough et al., 1987) facilitate the biological encoding of expectable environmental stimuli during constrained developmental windows, whereas experience-dependent stimuli are idiosyncratic processes that facilitate learning across the lifespan without ontogenetic constraints.

What we know better now (Reh et al., 2020) is that: (1) these windows occur for distinct domains and at different times over development; (2) the expected experience must coincide with the critical period for each circuit to occur; (3) plasticity is regulated at multiple timescales during development. The latter include different time-scaled processes: “(1) rapid, moment-by-moment shifts in circuit physiology; (2) gradual molecular events controlling the maturation of cortical circuits dictating critical period onset and closure in early life; and (3) epigenetic modifications over the life span (or across generations) that set the baseline level of plasticity” (Reh et al., 2020). Early environmental influences thus involve a complex development chronodependency of the child that opens up possibilities, if these CPs are given sufficient stimulation. Moreover, CPs are regulatory processes: they reduce future vulnerability to adversity, as experiences occurring after critical periods impact less brain circuitry (Takesian and Hensch, 2013). On the other hand, early adversity may modify CP processes, including their time of onset and closure.

This knowledge about CPs may help us to orient our intervention schedule (Nelson and Gabard-Durnam, 2020), especially in children experiencing social adversity (Nelson et al., 2019), or high-risk parenting (Feldman, 2015).

## Identifying children’s and parents’ strengths, and not only problems

Another assumption is that when we consider the target of intervention on a continuum from normal to pathological, we identify not only problems, but also individual strengths. This view is totally underestimated and underused in psychopathology. We are trained to detect what does NOT function, and not what the strengths are that we can lean on. A recent shift has therefore appeared in interventions targeting neurodevelopmental disorders. We have shifted from purely behavioral, early, intensive interventions, in a simplified environment, which aimed to decrease deviant behavior, to naturalistic developmental behavioral interventions, community based, peer-or parent-mediated (Schreibman et al., 2015), leaning on children’s and parents’ skills. These new interventions aim to address all the fields of normal development (cognition, motor development, language...), in order to improve the child’s engagement and reduce the vicious circle of unattuned interaction. In these interventions, the targets are normal developmental skills, and not pathological ones. Video feedback interventions with autistic children such as Preschool Autism Communication Therapy (PACT, Green et al., 2010), Video Feedback Intervention to promote Positive Parenting (VIPP, Poslawsky et al., 2014), and Interactive Guidance Therapy (IGT, Rusconi-Serpa et al., 2009) do not aim to teach new skills to the parents or to the child. They are based on the parent’s identification, in a co-construction with the therapist, of the respective interactive preserved skills along with the child during successful moments of interaction in short videotaped interactive play between parent and child. The conditions and occurrence of these moments are then generalized, decreasing the vicious negative interactive circle between the two partners. We do not work FOR the patient or the parent, but WITH them. We chose IGT (Ouss et al., 2023) instead of PACT, because it does not intervene at a specific symptom level, but at the intersubjective level. We determine in the here and now the tailored focus we will work on, depending on the actual play between the infant and the parent, and the parents’ responses to our questions. In PACT, the topic of each session is predetermined in advance by the method. This tenuous difference in the setting contains the core of what we consider the most important factor: the intersubjective link between the therapist and the patient or the parents. “Compared to the phenomenological approach, hermeneutic understanding is less unidirectional: it implies the co-construction of meaning and narratives in the course of the interactive process” (Fuchs, 2010). The early support of parents has recently developed as a fundamental trend in developmental psychopathology, becoming one of the four key points of interventions, together with the individualization of the intervention according to the developmental profile of the child, the expansion of learning targets, and the consideration of temporal characteristics (Wallace and Rogers, 2010).

## Conclusion

The extraordinary evolution of our models of psychopathology underlines the contributions of developmental and psychodynamic frameworks. These models make it possible to relativize the intuitive trend to focus our interventions on the most specific symptoms and syndromes, to the detriment of the uniqueness of each patient. These new models offer opportunities to reduce the “endogenous/exogenous dichotomy” in the mental health field. We have reached an era of “generalist” interventions in child psychopathology, which are paradoxically based on the ultrasingular of the idiographic dimension. We must assume that the clinician’s intuition is the guide of what we “feel,” and that this feeling is not noise to be eliminated, but rather the core guide of our interventions. The phenomenology of the clinician, mobilized through the interaction with the patient, no doubt needs to be conceptualized: does it refer to counter transference? Access to the unspeakable? The psychodynamic framework and psychoanalytical listening are probably the best tools to identify these dimensions, as has been shown in a very interesting research (Cohen et al., 2011). Instead of demonizing psychoanalysis and second-person perspectives, the richness and the limitations of current models of psychopathology, and the contribution of developmental approaches, should force us to reconsider our theoretical frameworks (Ouss-Ryngaert and Golse, 2010). These empirically informed directions are not yet in the mainstream of psychopathology frameworks, which prioritize Evidenced Based educational or behavioral based and guidelined interventions, as they are more straightforward to train. No guideline will teach how to listen to the singularity of each patient. The question: which skills a clinician must possess and how to equip them adequately with such skills remains a crucial concern. The paradox is that therapist’s non-specific skills, required for all types of psychotherapies (openness, attention to the patient’s world, non-judgment, empathy…) are not consistently incorporated into training, and are often regarded as inherent to a psychotherapist’s personality. We advocate that these skills should be developed during supervisions with experienced psychotherapists. Our proposals have to be developed to reach he deepest and the darkest corners of our patients, and of our own defenses.

## Data availability statement

The original contributions presented in the study are included in the article/supplementary material, further inquiries can be directed to the corresponding author.

## Author contributions

The author confirms being the sole contributor of this work and has approved it for publication.

## Footnotes

^1^ https://dictionary.apa.org/psychopathology
